# The Complex Spectrum of 11β-Hydroxylase Deficiency: A Case of Precocious Puberty, Hypertension, and Testicular Adrenal Rest Tumors (TARTs)

**DOI:** 10.7759/cureus.94192

**Published:** 2025-10-09

**Authors:** Naga Bhagyasri Mangam, Prasun Deb, Smitha Nalla, Sandeep Devireddy, Kiran Choudhary, Thushara Nayani

**Affiliations:** 1 Department of Endocrinology, Krishna Institute of Medical Sciences Hospital, Secunderabad, IND

**Keywords:** 11β-hydroxylase deficiency, congenital adrenal hyperplasia, cyp11b1 mutation, hypertension, precocious puberty, testicular adrenal rest tumours

## Abstract

11β‑hydroxylase deficiency (11β‑OHD), a rare form of congenital adrenal hyperplasia (CAH) accounting for roughly 1 in 100,000 live births, often presents with androgen excess and hypertension. It manifests in males as precocious puberty, often accompanied by hypertension, and in females as 46XX disorders of sex development with virilization. Long-term complications such as adult hypertension, impaired final height, and bilateral testicular adrenal rest tumors (TARTs) are infrequently reported.

We describe a 22-year-and-10-month-old male with a history of early-onset puberty beginning at 2.5 years of age, subsequent hypertension managed between the ages of 6 and 19, and medication noncompliance over the past three years. He presented with a two-year history of progressive bilateral testicular enlargement. Clinical assessment revealed stage 2 hypertension, short stature (height below the third percentile), generalized hyperpigmentation, and testicular enlargement with irregular surface. Biochemical evaluation showed elevated 11-deoxycortisol and 17-hydroxyprogesterone, an increased androstenedione:testosterone ratio, and suppressed cortisol-consistent with 11β‑OHD. Genetic testing confirmed a homozygous CYP11B1 mutation (c.1231G>C). Ultrasound detected bilateral testicular adrenal rest tumors. Treatment was initiated with hydrocortisone and spironolactone, leading to improved blood pressure control and reduced testicular size.

After three months of optimized steroid and antihypertensive therapy, blood pressure improved, testicular size decreased, and hormonal parameters showed partial normalization. Steroid dose adjustments were made to balance hypertension control with minimal bone and cardiovascular risks.

Management of 11β‑OHD requires careful titration of glucocorticoids, often necessitating higher doses than 21α-hydroxylase deficiency (21α‑OHD). Dexamethasone offers superior adrenocorticotropic hormone (ACTH) suppression but carries risks of cardiovascular morbidity and reduced bone density. It is particularly effective in treating TARTs and infertility. TARTs stages 1 to 3 respond to medical therapy; advanced stages may require surgical intervention, though fertility outcomes remain uncertain. Treatment must individualize goals based on patient age, pubertal status, fertility desires, and long-term cardiovascular and bone health.

This case highlights the importance of long-term follow-up in patients with 11β‑OHD, as noncompliance may predispose to complications such as hypertension and TARTs. Tailored therapy with glucocorticoids and antihypertensives can improve outcomes, though fertility preservation in the presence of advanced TARTs remains a clinical challenge.

## Introduction

Congenital adrenal hyperplasia (CAH) due to 11β‑hydroxylase deficiency accounts for 5%-8% of CAH cases and has an estimated worldwide incidence of 1 in 100,000 live births [1]. It is caused by mutations in the CYP11B1 gene, resulting in impaired conversion of 11‑deoxycortisol to cortisol and accumulation of mineralocorticoid precursors such as 11‑deoxycorticosterone, causing hypertension. Excess adrenal androgens cause virilization and rapid skeletal maturation. Males are also at risk of developing testicular adrenal rest tumors (TARTs), which may impair fertility. Long‑term follow‑up data into adulthood, with outcomes such as hypertension control and TART progression, remain limited.

## Case presentation

A 22-year-and-10-month-old male from Andhra Pradesh first presented in 2005, at the age of three years, with axillary and pubic hair development, penile enlargement, and generalized hyperpigmentation. His mother reported that pigmentation began at 2½ years of age, with no history of salt-wasting crises. By five years, he was the tallest in his class and exhibited acne, a deepened voice, increased appetite, and rapid weight gain compared with his peers. Testicular enlargement and scrotal pigmentation were noted by 5½ years of age.

He was started on dexamethasone 0.5 mg once daily at 10 p.m. and monthly GnRH analog injections due to advanced bone age and risk of central precocious puberty. His blood pressure normalized, pigmentation faded, and height velocity slowed. In 2014, he developed blurred vision, which was diagnosed as a posterior subcapsular cataract, leading to a temporary interruption of treatment. By 2018, the GnRH analog was discontinued, and testicular size increased.

From 2019 to 2024, he discontinued all medication. He re-presented in October 2024 with progressive testicular enlargement and skin hyperpigmentation over the previous two years.

Family history

**Figure 1:**
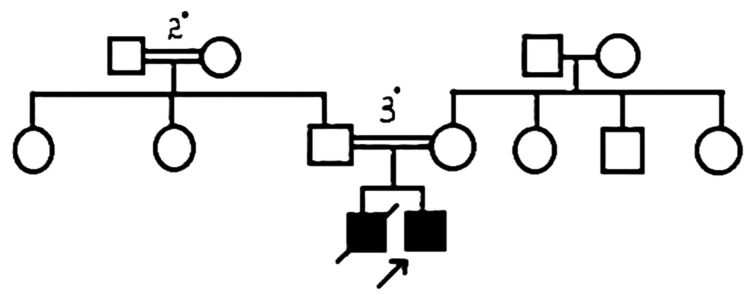
Pedigree chart. 2^o ^- second degree consanguinity, 3^o ^- third degree consanguinity.

The patient’s elder brother developed hyperpigmentation and secondary sexual characteristics at 2½ years of age. At three years, he experienced fever and loss of consciousness, was hospitalized, and subsequently passed away. Medical records were not available, but based on the history, possible causes included hypertension and neurological damage. The pedigree chart is shown in Figure 1. 

The patient’s father was diagnosed with diabetes mellitus at 46 years of age. There was no family history of early-onset hypertension, coronary artery disease, or cerebrovascular accident.

On examination

**Figure 2:**
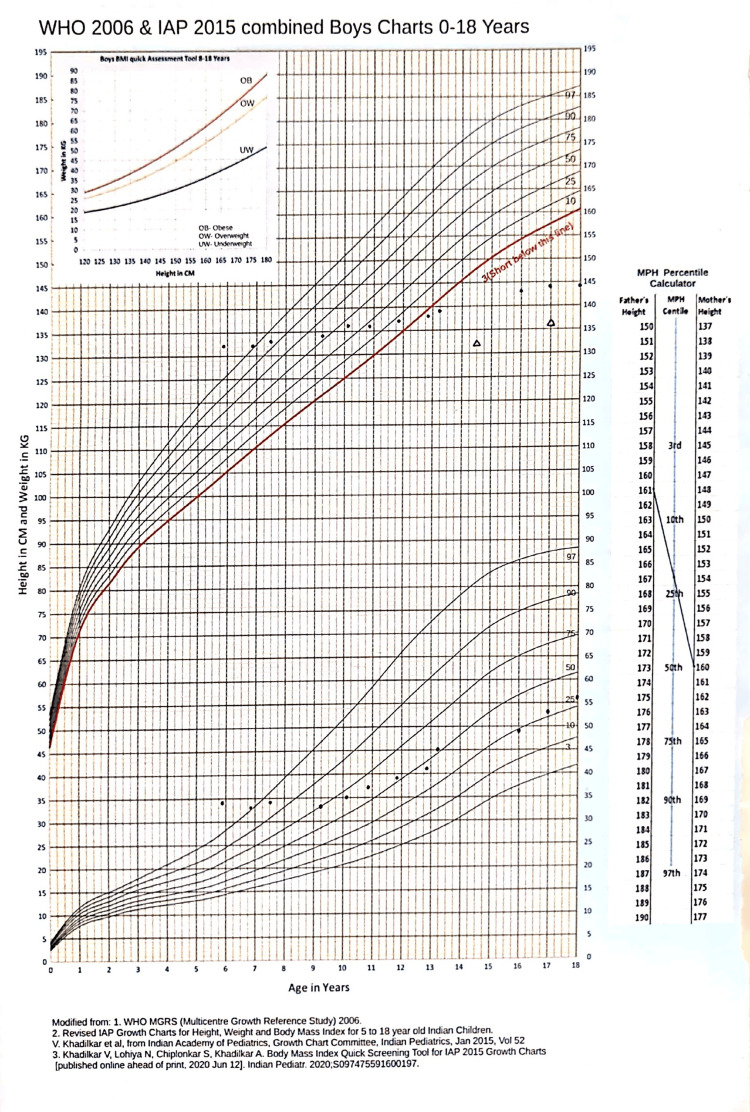
Growth chart. WHO-IAP, World Health Organization-Indian Academy of Pediatrics

The patient’s height was 144 cm, corresponding to -4.8 standard deviation score (SDS) at 18 years according to the World Health Organization-Indian Academy of Pediatrics (WHO‑IAP) growth chart (Figure 2). His weight was 56 kg, resulting in a body mass index (BMI) of 27 kg/m². Blood pressure measured 160/118 mmHg in the supine position and 156/116 mmHg when standing.

**Figure 3:**
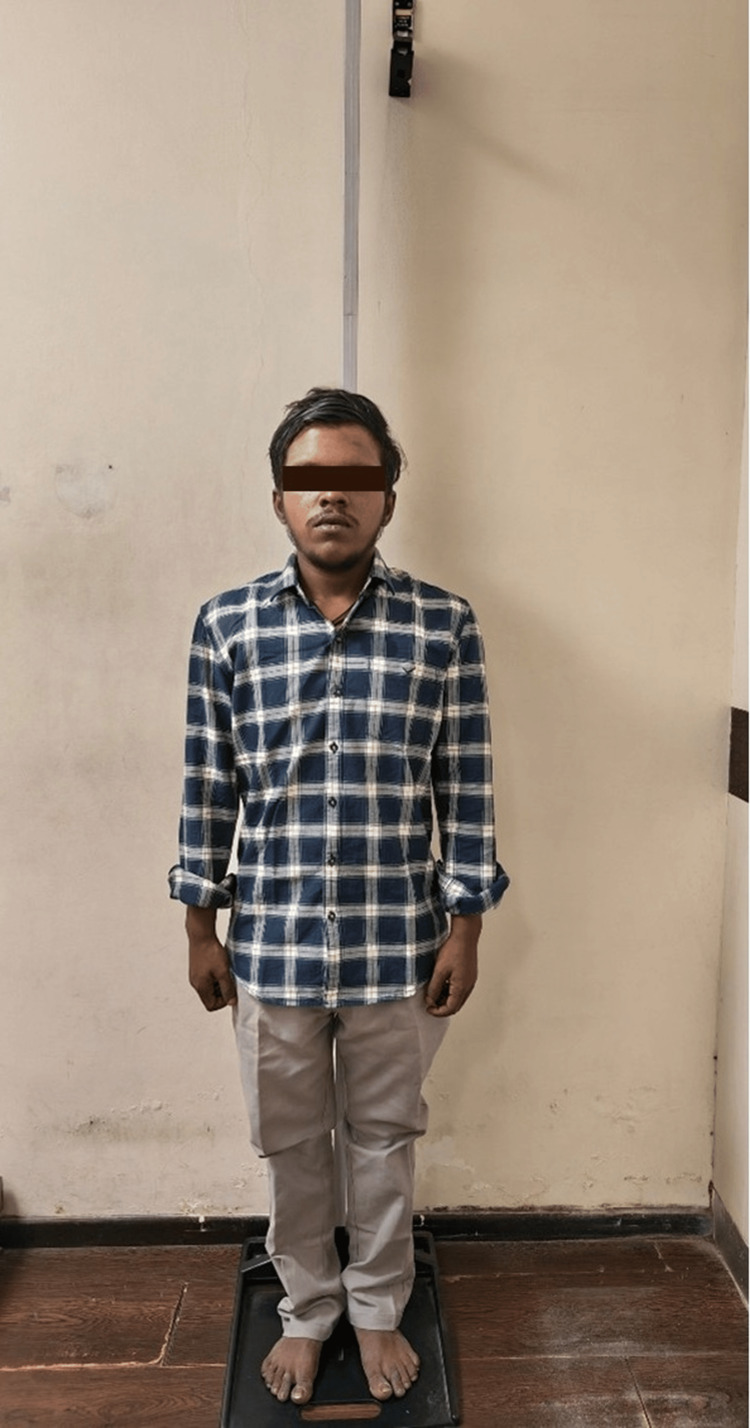
Picture showing short stature and hyperpigmented skin and lips.

**Figure 4:**
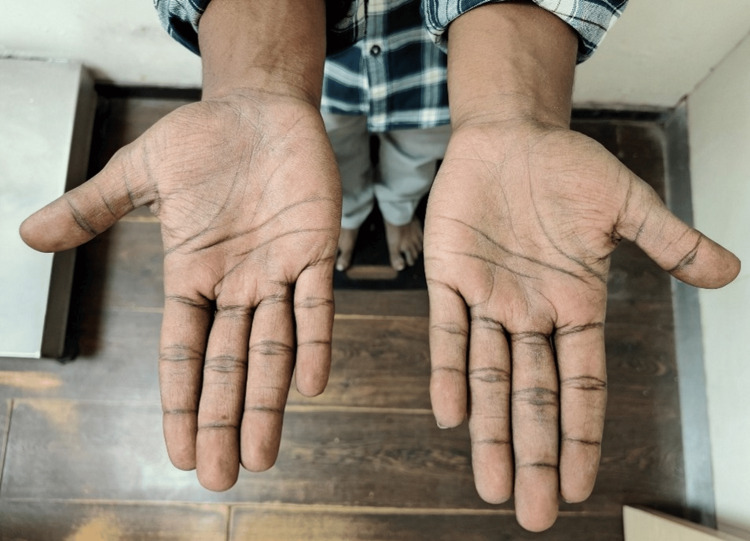
Hyperpigmented palms of the patient.

Diffuse hyperpigmentation of skin, lips, and palms is seen (Figures 3-4).

**Figure 5:**
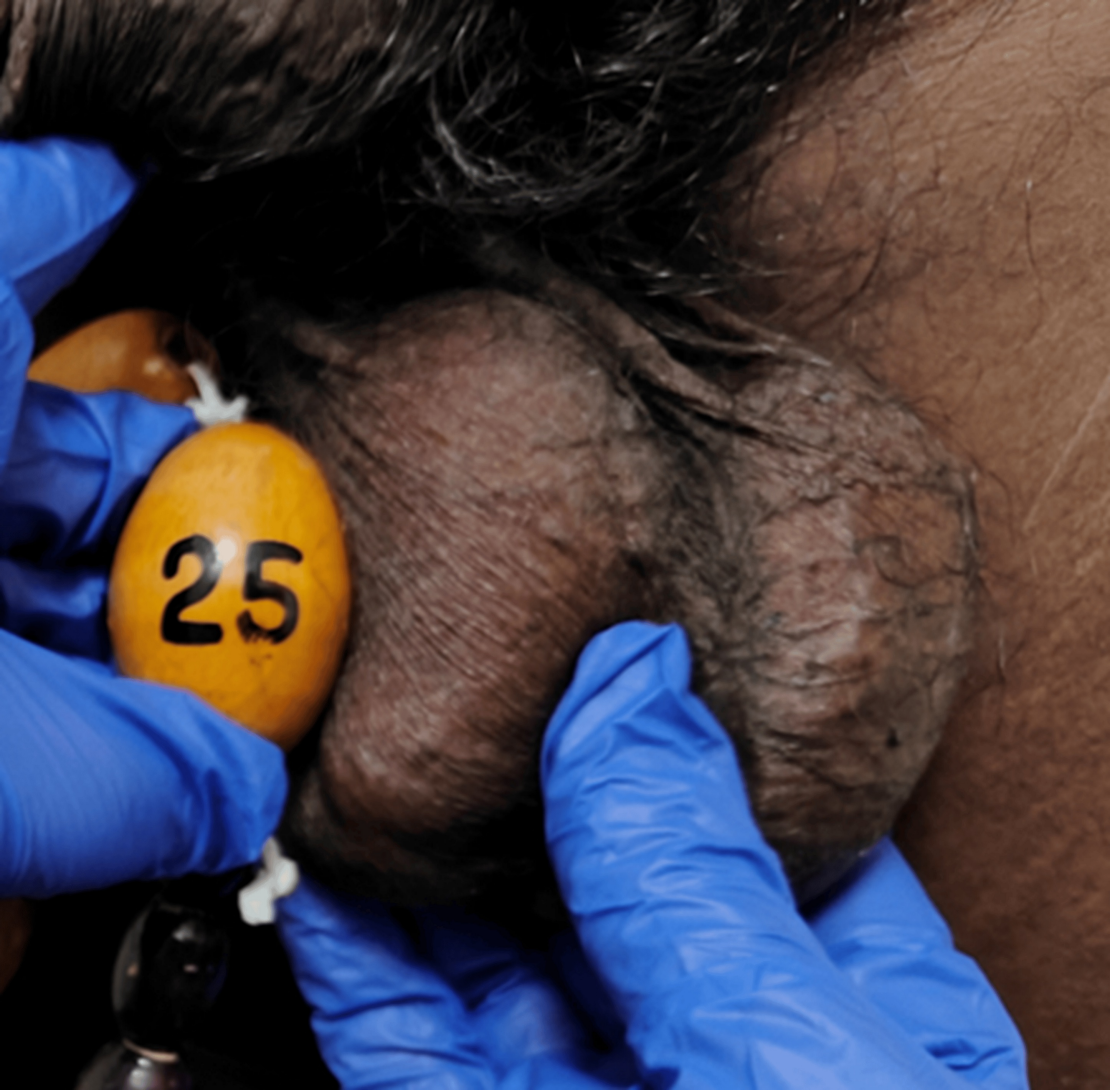
Bilateral enlarged testes.

Sexual maturity rating (SMR) was assessed as A+ P5. Stretched penile length (SPL) measured 10 cm. Testicular volume was greater than 25 cc bilaterally (left larger than right); the testes were firm and nodular, with prominent scrotal skin veins, as shown in Figure 5.

Examination revealed a posterior subcapsular cataract and grade 2 hypertensive retinopathy. Cardiac examination showed a loud second heart sound (S2) and a heaving apex, with no murmurs detected.

Investigations

Childhood hormonal profile showed low cortisol (3.21 µg/dL), markedly elevated adrenocorticotropic hormone (ACTH), elevated 17‑hydroxyprogesterone (17‑OHP), high-normal testosterone, and suppressed luteinizing hormone (LH) and follicle-stimulating hormone (FSH). Bone age was 14½ years at a chronological age of 5 years 10 months.

**Figure 6:**
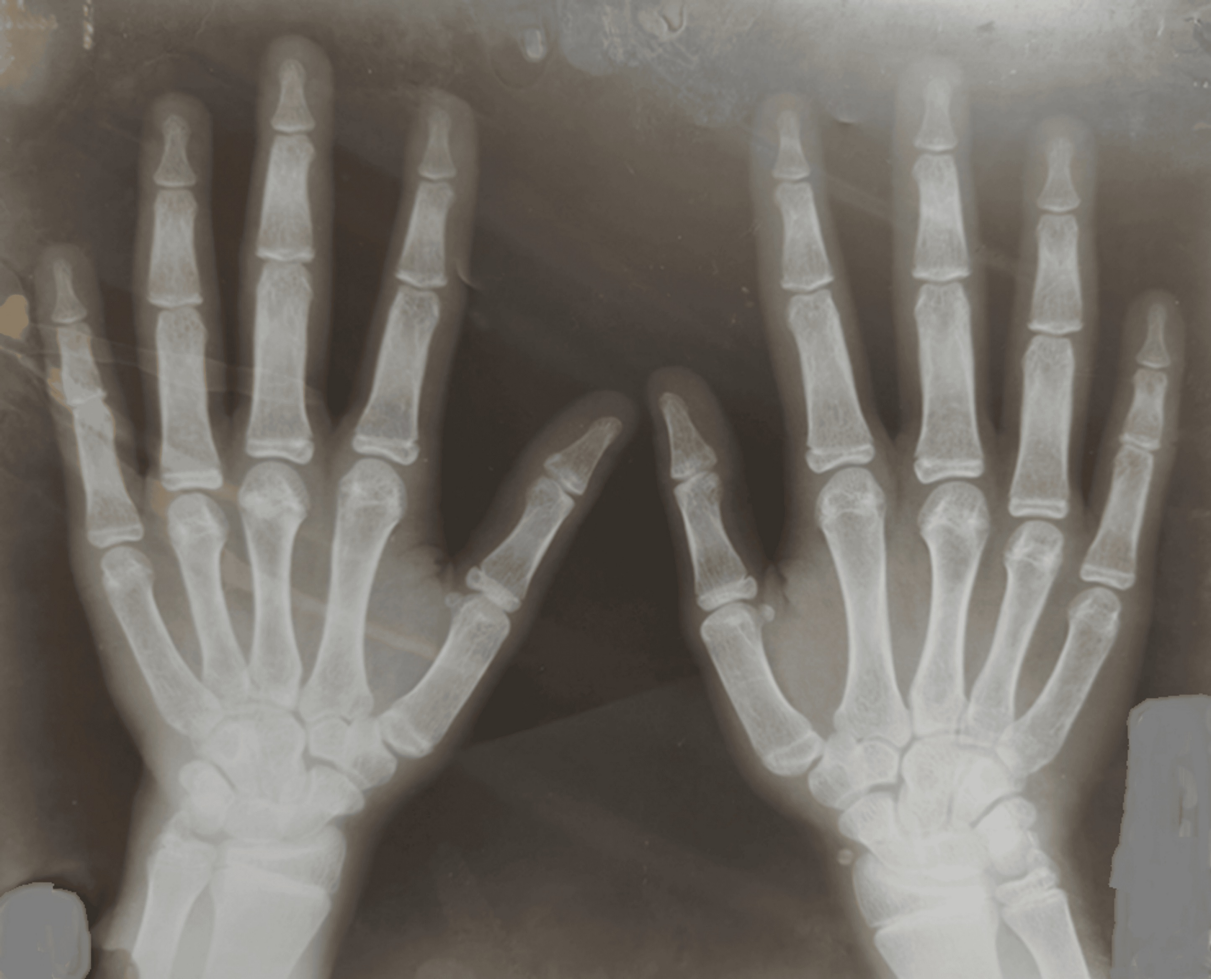
X-ray of both wrists and hands (posteroanterior view) at a chronological age of 10 years showing a bone age of 17 years.

An X-ray of both wrists and hands in posteroanterior (PA) view at 10 years of chronological age showed a bone age of 17 years, as shown in Figure 6. Height at 10 years was 136 cm, with a predicted adult height of 138.8 cm according to the Bayley and Pinneau chart.

Investigations performed in October 2024 at 22 years are summarized in Table 1. The liquid chromatography-mass spectrometry (LC-MS) steroid panel from the same time is shown in Table 2.

**Table 1:** Investigations in October 2024. FSH, follicle-stimulating hormone; LH, luteinizing hormone; Sr, serum

Parameter	Observed value	Reference range
Hb	15.2 gm/dL	13-17 gm/dL
Sr Sodium	145 mEq/L	135-145 mEq/L
Sr Potassium	2.8 mEq/L	3.5-5.1 mEq/L
Sr Creatinine	0.69 mg/dL	0.67-1.17 mg/dL
FSH	0.5 mIU/mL	1.72-19.26 mIU/mL
LH	0.7 mIU/mL	1.24-8.62 mIU/mL
Sr Testosterone	6.5 ng/mL	1.75-7.81 ng/mL
Urine protein	1+	Negative

**Table 2:** Liquid chromatography-mass spectrometry (LC-MS) steroid panel. DHEA, dehydroepiandrosterone; 17-OHP, 17‑hydroxyprogesterone; A:T, androstenedione:testosterone

Parameter	Observed value	Reference range
17-OHP	11.7 ng/mL	0.20-2.20 ng/mL
Cortisol	0.52 mcg/dL	Morning: 6.7-22.6 mcg/dL
11-Deoxycortisol	110 mcg/L (11,000 ng/dL)	0.5-3 mcg/L
11-Deoxycorticosterone	3.4 mcg/dL	0.02-0.15 mcg/dL
DHEA	0.9 mcg/L	
Androstenedione	36,600 ng/L	300-3100 ng/L
Testosterone	7.15 mcg/L	2.5-10 mcg/L
A:T	5.11	<0.5
21-deoxycortisol	0.01 mcg/L	0.02-0.15 mcg/L

Ultrasound examination revealed bulky adrenal glands and bilateral intratesticular hypoechoic vascular lesions near the mediastinum of the testes, measuring 33.1 cc on the right and 46.2 cc on the left, consistent with testicular adrenal rest tumors (TARTs).

Whole exome sequencing identified a homozygous CYP11B1 mutation (c.1231G>C, p.Gly411Arg), with autosomal recessive inheritance, suggestive of CAH due to 11β-hydroxylase deficiency (11β‑OHD).

Based on the clinical presentation and supporting investigations, a diagnosis of CAH due to 11β‑hydroxylase deficiency with bilateral TARTs was established.

The timeline of events is summarized in Table 3.

**Table 3:** Timeline of events showing the six-year period off treatment from 2018 to 2024, during which blood pressure (BP), testicular adrenal rest tumors (TARTs), and hypokalemia worsened. Lt, left; Rt, right

Parameter	15/4/2008	19/2/2009	14/7/2011	11/5/2015	27/4/2018	2/12/2018	30/10/2024
Testicular volume	Rt - 6 mL, Lt - 8 mL	Rt - 5 mL, Lt - 6 mL	Rt - 4 mL, Lt - 5 mL	Rt - 5 mL, Lt - 8 mL	Rt - 12 mL, Lt - 15 mL	Rt - 15 mL, Lt - 20 mL	Rt > 25 mL, Lt > 25 mL
BP (mmHg)	110/80	98/66	100/70	102/74	110/76	120/80	160/100
17-OHP (ng/mL)	9.87	-	-	-		-	11.7 ng/mL
DHEAS (µg/dL)	96.53	0.378	<0.10	0.9	-	-	-
Testosterone (ng/mL)	5.48	<0.08	0.2	0.6	-	4	7.15
Androstenedione (ng/L)	-	-	-	-	-	-	36,600
FSH (mIU/mL)	<0.05	-	-	-	-	12.6	0.5
LH (mIU/mL)	<0.05	-	-	-	-	4.9	0.7
Sr. cortisol (µg/dL)	3.21	-	<0.018	-	-	-	5.2
ACTH (pg/mL)	1261	-	-	26.91	88	-	-
Sr. potassium (mEq/mL)	-	-	-	-	-	3.3	2.8
Treatment advice	Tab. Dexamethasone 0.5 mg OD + Inj. Leuprolide 3.75 mg IM monthly	Tab. Dexamethasone 0.25 mg OD + continue leuprolide	Continue Dexamethasone 0.25 mg OD and leuprolide	Continue Dexamethasone 0.25 mg OD and leuprolide	Increase Dexamethasone to 0.5 mg and stop leuprolide	Continue dexamethasone (but the patient stopped medications and was lost to follow-up)	Started Tab. Hydrocortisone 10 mg at 8 a.m., 5 mg at 1 p.m., and 6 p.m. along with Spironolactone 50 mg BD + Amlodipine 5 mg

Management

In October 2024, the patient was started on hydrocortisone 20 mg/day, spironolactone 50 mg BID, and amlodipine 5 mg OD. In November 2024, the regimen was changed to hydrocortisone 5 mg at 8 a.m. and 1 p.m. plus dexamethasone 0.5 mg at 10 p.m., spironolactone 100 mg BID, and amlodipine 5 mg OD.

In May 2025, blood pressure was 120/80 mmHg, testicular volume was reduced clinically, and serum potassium was 4 mEq/L. The patient was continued on hydrocortisone 5 mg at 8 a.m. and 1 p.m., dexamethasone 0.5 mg at 10 p.m., spironolactone 50 mg, and amlodipine 5 mg OD.

**Figure 7:**
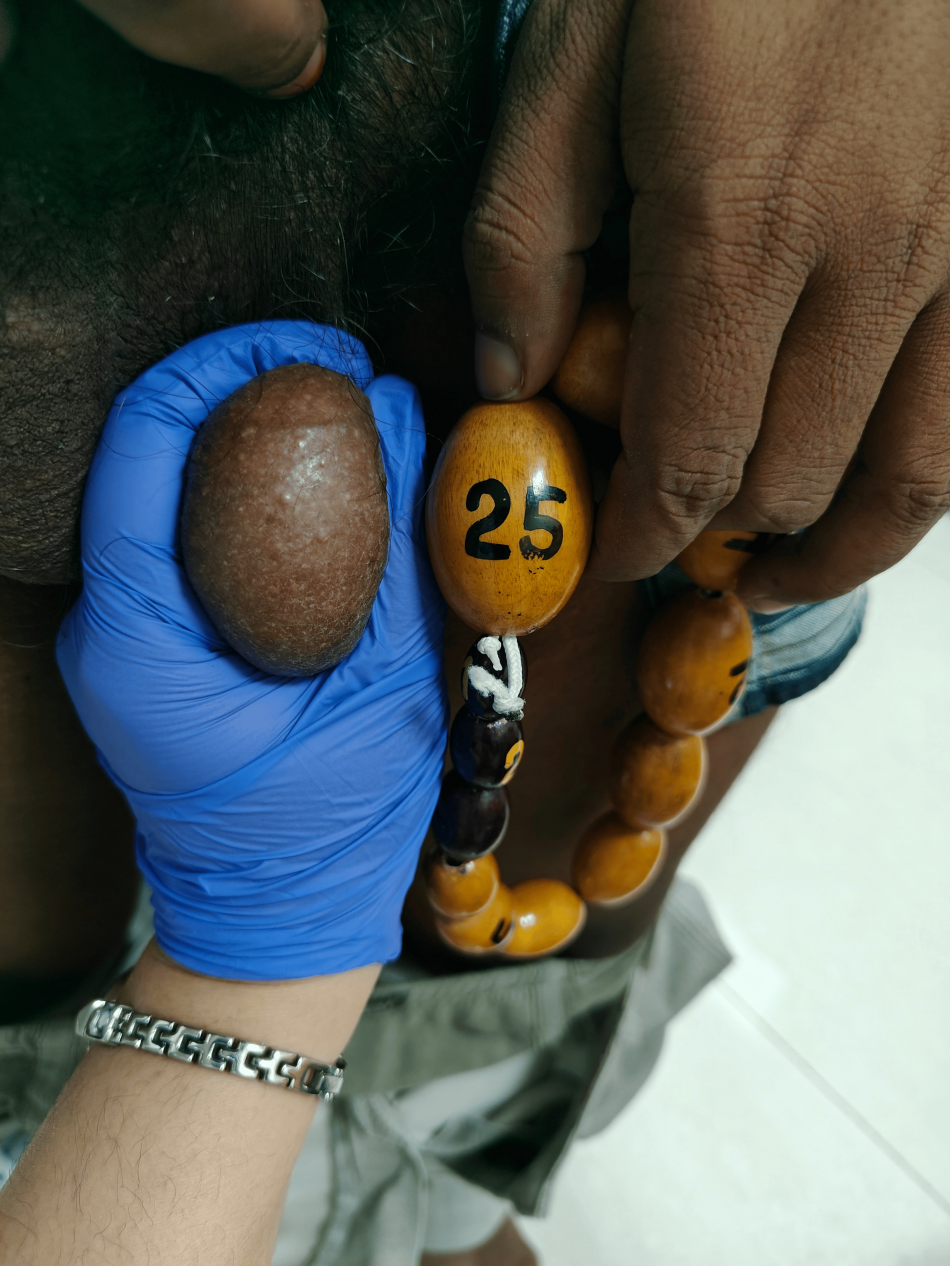
Follow-up testicular volume in September 2025.

In September 2025, blood pressure was 130/90 mmHg, with clinically reduced testicular volume (Figure 7). Laboratory parameters are shown in Table 4. The patient was continued on hydrocortisone 5 mg at 8 a.m. and 1 p.m., dexamethasone 0.5 mg at 10 p.m., spironolactone was stopped, and amlodipine was changed to 5 mg BD. 

**Table 4:** Laboratory investigations at follow-up. FSH, follicle-stimulating hormone; LH, luteinizing hormone; A:T, androstenedione:testosterone ratio

Parameter	16/5/2025	19/9/2025	Reference range
Potassium	4	4.9	3.5-5.1 mEq/L
FSH	o.51	16.4	1.72-19.26 mIU/mL
LH	0.7	9.2	1.24-8.62 mIU/mL
Testosterone	7.50	1.41	1.75-7.81 ng/mL
Androstenedione	>10	0.486	0.6-3.1 ng/mL
A:T ratio	-	0.34	<0.5

## Discussion

11β‑hydroxylase deficiency causes androgen excess, leading to peripheral precocious puberty and mineralocorticoid precursors‑induced hypertension. Advanced bone age at diagnosis limits adult height potential. TARTs occur in up to 94% of adult males with poorly controlled CAH and respond to ACTH suppression with steroids when detected in early stages.

The androstenedione-to-testosterone (A:T) ratio helps distinguish between testicular and adrenal sources of androgens. An A:T ratio greater than 0.5 suggests an adrenal origin, as seen in active CAH, whereas a lower ratio indicates that the testes are the primary source.

In our case, an A:T ratio of 5.11 (markedly elevated) reflects severe, uncontrolled adrenal androgen excess. This supports both the diagnosis and the need for intensified ACTH suppression.

This case illustrates the importance of adherence to glucocorticoid therapy for long-term disease control. It also demonstrates the potency of dexamethasone in suppressing ACTH and promoting regression of TARTs, along with its potential risks, including iatrogenic Cushing’s syndrome, cardiovascular morbidity, and reduced bone mineral density (BMD). The pros and cons of dexamethasone are summarized in Table 5.

**Table 5:** Pros and cons of dexamethasone in 11‑beta-hydroxylase deficiency congenital adrenal hyperplasia (CAH) with testicular adrenal rest tumors (TARTs). ACTH, adrenocorticotropic hormone; BMD, bone mineral density

Pros	Cons
30x more potent in ACTH suppression compared to hydrocortisone	80x more growth retarding effect compared to hydrocortisone
Treating TARTs	Higher risk of Iatrogenic Cushing’s syndrome and cardiovascular diseases
Infertility	Decreased BMD

This underscores the necessity of multiple antihypertensives for blood pressure control, particularly mineralocorticoid antagonists and calcium channel blockers [2-9].

We differentiated this from glucocorticoid resistance [10] based on very low cortisol and high ACTH levels with suppressed gonadotropins, consistent with classic 11β‑hydroxylase deficiency biochemistry, and the presence of a pathogenic CYP11B1 mutation.

TARTs are benign intratesticular lesions arising from aberrant adrenal cortical cells that persist along the gonadal descent pathway and proliferate under chronic ACTH stimulation, especially in poorly controlled CAH, but can also occur in well-controlled CAH [11]. In 11β‑OHD, persistent ACTH drive from inadequate cortisol feedback promotes both adrenal hyperplasia and TART development. They originate from ectopic adrenal-like cells located in the rete testis, stimulated chronically by elevated ACTH. Lack of cortisol in 11β‑OHD leads to sustained ACTH hypersecretion; elevated ACTH mediates hypertrophy and proliferation of these adrenal rest cells. They are usually bilateral, adjacent to the mediastinum testis, mimicking Leydig cell tumors on imaging. TARTs are a major cause of infertility in adult males with CAH. Tumor expansion compresses seminiferous tubules, causing obstructive azoospermia and damage to Leydig and Sertoli cells. Early detection (Stages 1-3) allows reversibility with optimized glucocorticoid therapy, restoring gonadal function and preserving fertility. Late stages (Stages 4-5) lead to irreversible fibrosis and permanent infertility.

Regular follow-up with annual testicular ultrasound, semen analysis (for adults), and hormonal profiling is recommended. Surgical intervention is reserved for non-responsive or fibrotic late-stage TARTs but may not restore fertility. Long-term outcome hinges on early detection, adherence to steroid therapy, and meticulous balancing between ACTH suppression and systemic side effects.

## Conclusions

Advanced bone age at diagnosis limits final height; therefore, treatment should focus on effective androgen control and prevention of central precocity. In 11β‑OHD, higher glucocorticoid doses are often required compared to 21α-hydroxylase deficiency (21α‑OHD).

By aggressively suppressing ACTH using dexamethasone, the adrenal androgen excess and mineralocorticoid effects were curtailed, resulting directly in regression of TARTs and normalized BP.

Dexamethasone achieves the clinical goal but must be balanced against side effects, including iatrogenic Cushing’s syndrome and decreased bone density.

TARTs respond well to medical therapy in the early stages, while late-stage disease becomes irreversible. Although dexamethasone is effective in suppressing TARTs, its use is limited by significant long-term risks. Regular follow-up with BP monitoring, serum potassium assessment, gonadal imaging, and hormonal profiling is essential for optimal management.
